# Mycotoxin management in the cereal supply chain: Advanced detection, detoxification, and smart packaging strategies

**DOI:** 10.1002/jsfa.70739

**Published:** 2026-06-05

**Authors:** Abolfazl Asqardokht‐Aliabadi, Dipendra Kumar Mahato, Jafar Mohammadzadeh Milani

**Affiliations:** ^1^ Department of Food Science and Technology Sari Agricultural Sciences and Natural Resources University Sari Iran; ^2^ CASS Food Research Centre, School of Exercise and Nutrition Sciences Deakin University Burwood VIC Australia

**Keywords:** mycotoxin, cereal grain, rapid detection, nanotechnology, biotechnological detoxification, smart packaging

## Abstract

Mycotoxins remain a persistent threat to the safety and quality of cereal grains and other agricultural products, and their impact on human health continues to raise global concerns. In many situations, the practices traditionally used to control these toxins are no longer sufficiently effective. They can be costly, difficult to implement on a large scale, and, in some cases, create environmental drawbacks. As cereal products move through the supply chain, the need for faster and more sensitive detection tools, more reliable detoxification options, and practical postharvest preventive measures has become increasingly clear. This review scrutinizes recent advances in biotechnology‐ and nanotechnology‐based approaches, focusing on their superior environmental sustainability compared to classical methods. Specifically, it evaluates how nanoparticle‐assisted biosensors and advanced spectroscopic techniques significantly enhance the sensitivity and selectivity of mycotoxin monitoring systems. Biological and nanocatalytic detoxification methods, which provide milder and more environmentally friendly modes of action, are also discussed. In addition, advances in active and smart packaging are considered, especially their potential to limit fungal growth and prevent recontamination during storage and distribution. Taken together, these developments suggest that combining high‐performance analytical tools with emerging bio–nano solutions could support more effective and sustainable mycotoxin management throughout the cereal supply chain. © 2026 The Author(s). *Journal of the Science of Food and Agriculture* published by John Wiley & Sons Ltd on behalf of Society of Chemical Industry.

**ABBREVIATIONS**
AF: Aflatoxins
AFB₁: Aflatoxin B₁
AI: Artificial intelligence
ANN: Artificial neural network
BsDyP: Dye‐decolorizing peroxidase
C₁₈: Octadecyl silica
CNN: Convolutional neural network
DAD: Diode array detector
DON: Deoxynivalenol
ECD: Electron capture detector
E‐nose: Electronic nose
ELISA: Enzyme‐linked immunosorbent assay
FB: Fumonisin B
FID: Flame ionization detector
FPIA: Fluorescence polarization immunoassay
FRET: Förster (fluorescence) resonance energy transfer
FTIR: Fourier transform infrared
FUM: Fumonisins
FumD: Fumonisin esterase
GC: Gas chromatography
GCB: Graphitized carbon black
GPS: Global positioning system
HACCP: Hazard analysis and critical control points
HPLC: High‐performance liquid chromatography
HRMS: High‐resolution mass spectrometry
HSI: Hyperspectral imaging
IoT: Internet of Things
LC–MS/MS: Liquid chromatography–tandem mass spectrometry
LCC: Life‐cycle cost
LFIA: Lateral flow immunoassay
LOD: Limit of detection
LOQ: Limit of quantification
MIR: Mid‐infrared
MIP: Molecularly imprinted polymer
ML: Machine learning
MS: Mass spectrometry
NIR: Near‐infrared spectroscopy
NMR: Nuclear magnetic resonance
PSA: Primary secondary amine
QuEChERS: Quick, easy, cheap, effective, rugged, and safe
RFID: Radio‐frequency identification
SERS: Surface‐enhanced Raman spectroscopy
SIDA: Stable isotope dilution analysis
SPE: Solid‐phase extraction
SVM: Support vector machine
TLC: Thin‐layer chromatography
TRL: Technology readiness level
UCNP: Upconversion nanoparticle
UHPLC: Ultrahigh‐performance liquid chromatography
UV: Ultraviolet
Vis–NIR: Visible–near‐infrared
XGB: Extreme gradient boosted trees
ZEN: Zearalenone
Zhd101p: Zearalenone‐lactonase

## INTRODUCTION

Mycotoxins are toxic secondary metabolites naturally produced by filamentous fungi, mainly species belonging to the genera *Aspergillus*, *Penicillium*, and *Fusarium*. These compounds frequently contaminate a wide range of agricultural commodities and represent a major global threat to food and feed safety.^1^, ^2^ Classical estimates from FAO suggested that around 25% of global cereal production exceeded safety thresholds for mycotoxins; however, more recent monitoring data indicate that 60–80% of cereal‐based products may contain detectable levels, although concentrations often remain below regulatory limits depending on region, commodity, and season.^3^, ^4^

The observed increase in reported contamination can be attributed both to improved analytical sensitivity and to shifts in fungal ecology associated with global climate change. Among the most prevalent contaminants in cereals such as maize, wheat, barley, and oats are Aflatoxin B_1_ (AFB_1_), deoxynivalenol (DON),^5^ fumonisins (FUM),^6^ and zearalenone (ZEN),^7^ which are known to cause severe health issues in both humans and animals, including liver and nervous system damage, immune suppression, and carcinogenic effects.^1^ Beyond single‐toxin contamination, co‐occurrence of multiple mycotoxins has emerged as a major concern. Recent surveys suggest that up to 60% of cereal samples may contain two or more mycotoxins, frequently combinations of DON, ZEN, and FUM, complicating toxicological assessment and risk management.^8^, ^9^

Another significant complication is the heterogeneity of mycotoxin distribution between lots of cereals. Contamination usually occurs in local ‘hot spots’, which makes representative sampling problematic and leads to higher risks of underestimation or false negatives during monitoring.^10^, ^11^ Traditional methods for mycotoxin control, including physical sorting, heat treatments, and chemical elimination, have proven ineffective and often face major drawbacks.^12^ In many cases, the toxins present may be in an indigestible form, which could result from hydrolysis or degradation by a relevant enzyme; therefore, these procedures might not be entirely effective and could also worsen the nutritional value or sensory properties of the food.^13^ Such limitations have imposed the urgency to shift from conventional methods to more viable, selective, and eco‐friendly means.^12^ Recent advancements in science and technology, particularly in biotechnology, nanotechnology, and materials science, have significantly transformed the detection and management of mycotoxins.^14^

Several review papers have recently examined different facets of mycotoxin contamination in cereal grains. Hamad *et al*.^15^ concentrated on emerging control strategies, including cold plasma, polyphenols, and nanoparticles. In contrast, Girmatsion *et al*.^16^ and Mustafa *et al*.^17^ focused on detection technologies, specifically machine‐learning‐assisted spectroscopy and electronic nose (E‐nose). Niaz *et al*.^18^ offered an updated account of chromatographic methods for mycotoxin analysis in cereals. Although these contributions are valuable, each remains confined to a single domain, either detection or detoxification, and none adopts an integrated, supply‐chain‐wide perspective. More importantly, no previous review has systematically combined the three core pillars of (i) advanced detection methods suitable for on‐farm or in‐line use, (ii) biological and nanocatalytic detoxification technologies, and (iii) smart packaging as a unified strategy. The present review fills this gap by introducing, for the first time, a convergent approach that spans the entire cereal supply chain. It demonstrates how the synergy among these three areas can enable more effective, sustainable, and real‐time mycotoxin management from farm to fork.

Apart from the public health dimension, mycotoxin contamination results in significant economic issues due to the downgrading of grain to animal feed, product recalls, disposal costs, and trade restrictions. Such economic expenses are especially pertinent in export‐oriented cereal supply chains.^9^, ^19^ This review provides an in‐depth and supply‐chain‐oriented overview of advanced methods for controlling mycotoxins. Three interrelated pillars are highlighted: (i) novel detection techniques with high analytical sensitivity and on‐site convenience, (ii) advanced detoxification technologies based on biological, enzymatic, and nanocatalytic processes, and (iii) smart packaging systems that are capable of preventing recontamination at the time of distribution, transport, and storage. Through synergies in advances in these domains, the review here outlines the way new technologies can bridge the gaps in existing management practice and enable mycotoxin management to be sustainable across the worldwide cereal supply chain.

## ADVANCED DETECTION METHODS

Although chromatographic methods such as high‐performance liquid chromatography (HPLC) and gas chromatography (GC), as well as liquid chromatography–tandem mass spectrometry (LC–MS/MS), remain the gold standard for quantitative and regulatory analysis, the industrial demand for rapid, inexpensive, and on‐site methods has pushed the development of innovative complementary approaches to this end.^20^ At the laboratory level, surface‐enhanced Raman spectroscopy (SERS) platforms and electrochemical nanosensors provide ultrahigh sensitivity in the range of picograms to nanograms and allow integration with sample pretreatment methodologies (e.g. quick, easy, cheap, effective, rugged, and safe (QuEChERS) or magnetic solid‐phase extraction (SPE) to avoid matrix effects).^21^, ^22^, ^23^

Sample preparation techniques such as modified QuEChERS, magnetic SPE, and stable isotope dilution analysis (SIDA) play a backbone role in minimizing matrix interference and in improving the qualitative/quantitative reliability of an assay.^21^, ^24^ In terms of data analytics, coupling high‐resolution mass spectrometry (HRMS) outputs with artificial intelligence (AI) and metabolomics will bring about greater prospects in detecting ‘masked/conjugated’ mycotoxins and new metabolites and derivative forms.^18^

To successfully translate these technologies to industrial setups, each method must be reported with standardized validation parameters (limit of detection (LOD) and limit of quantification (LOQ), linearity, recovery, repeatability, and interlaboratory testing) and with the use of appropriate reference materials to ensure regulatory acceptance and market confidence.^24^, ^25^ Finally, the technology readiness level (TRL), life‐cycle cost (LCC), and safety aspects connected with the use of a given nanomaterial should preferably be reported along with performance data to allow both readers and decision‐makers to assign a level of suitability to the technologies concerning either the field or laboratory environment.^26^

Conventional chromatographic methods offer the foundation of mycotoxin measurement of cereal‐derived products.^27^ HPLC, in combination with ultraviolet (UV), diode array, fluorescence, or MS detectors, is the common method of choice.^28^ GC, with electron capture, flame ionization, or MS detectors, is utilized mostly to identify mycotoxins.^29^ Among different methods, LC–MS/MS is an increasingly popular method of simultaneous screening, identification, and measurement of multiple mycotoxins.^30^

Sample preparation is required to determine entities of trace concentrations accurately. Traditional methods comprise SPE and specialized cleanup columns, including multifunctional columns like MycoSep® and immunoaffinity columns.^24^, ^31^ Complex methods, of which QuEChERS is a representative, enable simultaneous multianalyte detection of multiple mycotoxins within intricate matrices by multiple layers of composite cleanup procedures and are useful in various materials, including cereals, spices, nuts, noodles, eggs, human biological fluids, and baby food.^32^ Moreover, auxiliary purification steps involving sorbents, including octadecyl silica (C_18_), primary secondary amine (PSA), and graphitized carbon black (GCB), are typically incorporated within the QuEChERS method to eliminate/deplete matrix interferences and achieve maximal percentages of recovery.^33^

Immunometric assays, and specifically enzyme‐linked immunosorbent assays (ELISAs), continue to be utilized extensively for general screening purposes.^34^ Although commercially sourced ELISA kits offer rapid analysis, their cost is due to the necessity of unique antibodies specifically developed for each target mycotoxin.^25^ Alternative older methods include thin‐layer chromatography (TLC) and spectroscopic procedures, such as Fourier transform infrared (FTIR) and nuclear magnetic resonance (NMR) spectroscopies.^35^ Although analytically these procedures possess higher precision, they suffer from being tied to considerations of their demands on time, costs, instrumental sophistication, and their unsuitability for large‐scale or on‐site monitoring.^36^, ^37^

### Masked mycotoxins: analytical and toxicological challenges

The issue of masked mycotoxins was brought to light in the 1980s, when there were inexplicable instances of mycotoxicity in animals, although toxins in feed were only detected at a very low level.^6^, ^38^ Masked mycotoxins refer to glucose‐conjugated derivatives of fungal mycotoxins that cannot be detected by normal analysis due to structural modification.^39^, ^40^ Masked mycotoxins result from an enzymatic reaction in which plants conjugate glucose, sulfate, or amino acids as a defense mechanism against mycotoxins, leading to the formation of more polar derivatives such as DON‐3‐glucoside and ZEN‐14‐glucoside.^7^, ^41^

Their analysis poses a difficult challenge due to the influence of changed polarity and structure on the processes of extraction and chromatographic separation.^39^ LC–MS/MS analysis techniques involving various hydrolysis procedures are the most commonly used approach despite the absence of analysis standards and the complexity of isomers that often create difficulties in precise quantification analysis.^38^, ^42^ From the toxicological perspective, these conjugated substances can undergo hydrolysis either during digestion or processing to release the original toxin and hence recover toxicity.^41^, ^43^ Despite the important role they play, masked or chemically modified mycotoxins do not have specific levels set by regulation against them in estimates of risk assessment and exposure analysis.^38^, ^44^

### Spectroscopic and imaging techniques

Spectroscopic methods are now significant alternatives to chromatographic methods due to their nondestructive, fast, and minimally preparative features. Techniques including near‐infrared (NIR), visible–NIR (Vis–NIR), mid‐infrared (MIR), fluorescence spectroscopy, hyperspectral and multispectral imaging, and SERS facilitate the rapid screening of cereals for contamination with mycotoxins in wheat, maize, rice, sorghum, and corn.^22^, ^26^, ^45^, ^46^ The combined use of SERS and QuEChERS has high sensitivity and specificity, achieving detection limits as low as 0.85 μg kg^−1^ for aflatoxin B_1_.^22^

#### Hyperspectral imaging (HSI) coupled with machine learning (ML)

Unlike conventional technologies, when combined with ML models, HSI technology can be used to assess and classify mycotoxins in grains and nuts by detecting spectral signatures unique to the samples under study. Recent studies have demonstrated high prediction accuracy and the selection of relevant wavelength bands for training classification algorithms.^47^ For example Kim *et al*.^48^ used HSI for simultaneous detection of aflatoxin B_1_ and FUM in ground maize with high precision. Similarly, Williams *et al*.^49^ applied ML techniques to hyperspectral images and achieved 96.67% classification accuracy for aflatoxin infection in pistachio nuts.

### Molecularly imprinted polymers and nanomaterials

Molecularly imprinted polymers (MIPs) can mimic the recognition properties characteristic of biological antibodies and can be applied to achieve selective mycotoxin enrichment.^50^ The combination of MIPs with SPE methods, along with electrochemical sensors, fluorescent‐based sensors, SERS, and surface plasmon resonance platforms, has achieved very specific and sensitive detection capabilities.^51^ Sensors based on nanomaterials with graphene oxide and polymer–metal–organic system hybrids have been applied to amplify signals, to bind biomolecules, and to attain Förster (or fluorescence) resonance energy transfer (FRET)‐based mycotoxin identification.^22^, ^52^, ^53^ Nanohybrid materials have been applied as new detoxifying agents too; that is, *β*‐glucan mannan lipid particles encapsulating humic acid nanoparticles that yield effective aflatoxin B_1_ adsorption during their application to feeds.^52^

### Advanced sample preparation methods

Besides conventional SPE and the QuEChERS method, magnetic SPE using magnetic nanoparticles or GCB can be a rapid and efficient way to separate mycotoxins from actual diet matrices.^23^ The combination of QuEChERS with UHPLC–MS/MS allows simultaneous measurement of regulated and emerging mycotoxins such as the modified *Alternaria* and *Fusarium* toxins.^21^, ^54^ Despite this, the detection systems that are most advanced can still just indicate and measure the risk; they do not automatically remove or stop mycotoxin contamination. Hence, the next vital action for getting total control is putting in place, through the cereal supply chain, the most effective detoxification and preventive measures. Wu *et al*.^55^ successfully generated the first polyclonal antibodies and oligonucleotide aptamers specifically targeting fusaric acid. They developed three highly sensitive assays (ELISA, ELASA, and a hybrid ELAAA) with detection limits ranging from 5 to 50 ng g^−1^ in cereal samples, achieving recoveries of 87–112%.

### Emerging rapid and portable detection technologies

#### Microfluidic lab‐on‐a‐chip systems

Lab‐on‐chip devices combine sample extraction, separation, and detection all in a small, integrated platform for rapid applications with minimal reagent usage. Their high throughput and portability make them ideal for on‐site testing at storage facilities and grain processing facilities.^19^ They also can have an immunoassay, nanomaterial, or electrochemical detection module for added sensitivity and selectivity.

#### Single kernel analysis

With technologies that use fluorescence imaging, reflectance spectroscopy, and LC‐based microscreening, it is possible to conduct assessments for mycotoxins directly at the individual kernel level. Individual kernel assessments increase accuracy by allowing for better identification of contamination within a batch and reducing the likelihood of false negatives associated with bulk sampling, while also providing additional benefits with the sorting remediation tools that are available.^19^ Single kernels act as a particularly useful resource for identifying localized contamination ‘hot spots’.^56^

#### E‐nose platforms

E‐noses utilizing sensor arrays of metal oxide semiconductors have been developed for the detection of volatile organic compounds, which may indicate the growth of fungi. Three‐parameter models of impregnation time, cleaning time, and maximum sensor response are more practical in terms of detecting organic compounds for a rapid screening of stored cereals.^57^, ^58^ Their nondestructive nature and applicability within storage settings support an early intervention. For example, Sebti *et al*.^59^ developed an AI‐enhanced Internet of Things (IoT)‐based E‐nose that detected ochratoxin A in maize with >98.5% accuracy within 1 s.

#### Fluorescence polarization immunoassay (FPIA)

FPIAs allow quick and low‐reagent screening and do not require extensive sample cleanup. The assay depends on a competitive binding mechanism and on the detection of polarized light to generate semiquantitative data within some minutes.^60^, ^61^ The low cost and adaptability of these assays to portable readers make them suitable for routine work. Although FPIA is a well‐established technique, recent innovations have expanded its applicability. For instance, Wu *et al*.^55^ developed novel antibody‐ and aptamer‐based assays (ELISA, ELASA, and ELAAA) for the sensitive detection of fusaric acid in cereals, achieving limits of detection as low as 5–50 ng g^−1^ with recoveries of 87–112%. These approaches demonstrate the power of immunoassay platforms for rapid mycotoxin screening.

### Scalability and industrial integration

Several emergent technologies, such as microfluidic chips, handheld infrared spectrometers, biosensor‐equipped screw feeders, and HIS systems, show scalability prospects in industrial applications.^62^ Transportation and storage‐stage screening can be achieved rapidly with handheld spectroscopic devices,^63^ while biosensor‐based systems can be directly incorporated on processing lines so that real‐time monitoring can be achieved with immediate corrective actions.^64^, ^65^ Development as well as scalability of these approaches rests on demonstration against requirements by regulators, economies, and amenability towards scalable high‐throughput processing.

While extremely sophisticated detection technologies greatly enhance the identification and detection of mycotoxins, detection works no better at preventing or eliminating the threats they pose throughout the food chain than it does at identifying and tracing them. The next essential step towards comprehensive control of mycotoxins involves applications of detoxification and deactivation technologies, which are discussed in the following section.

## INNOVATIVE DETOXIFICATION STRATEGIES

Conventional approaches to mycotoxin management frequently prove inadequate, given the high resistance of many mycotoxins to standard treatments. For example, while physical methods like heat treatment, such as cooking, baking, roasting, and extrusion, are commonly utilized in industrial food processing, these techniques often do not fully eradicate contamination. Mycotoxins frequently present in cereals, such as aflatoxins, FUM, and deoxynivalenol, demonstrate significant thermal stability.^66^ Industrial techniques often decrease mycotoxin levels in food considerably; however, they do not eliminate them. Therefore, completely processed meals may still present risks.^67^ Relying only on heat treatment for disinfection has many drawbacks since certain techniques like alkaline cooking (nixtamalization) and corn flake manufacture have inconsistent and often inadequate impacts on antibacterial stability.^66^ The main groups of detoxification approaches discussed in this section are schematically summarized in Fig. 1.

**Figure 1:**
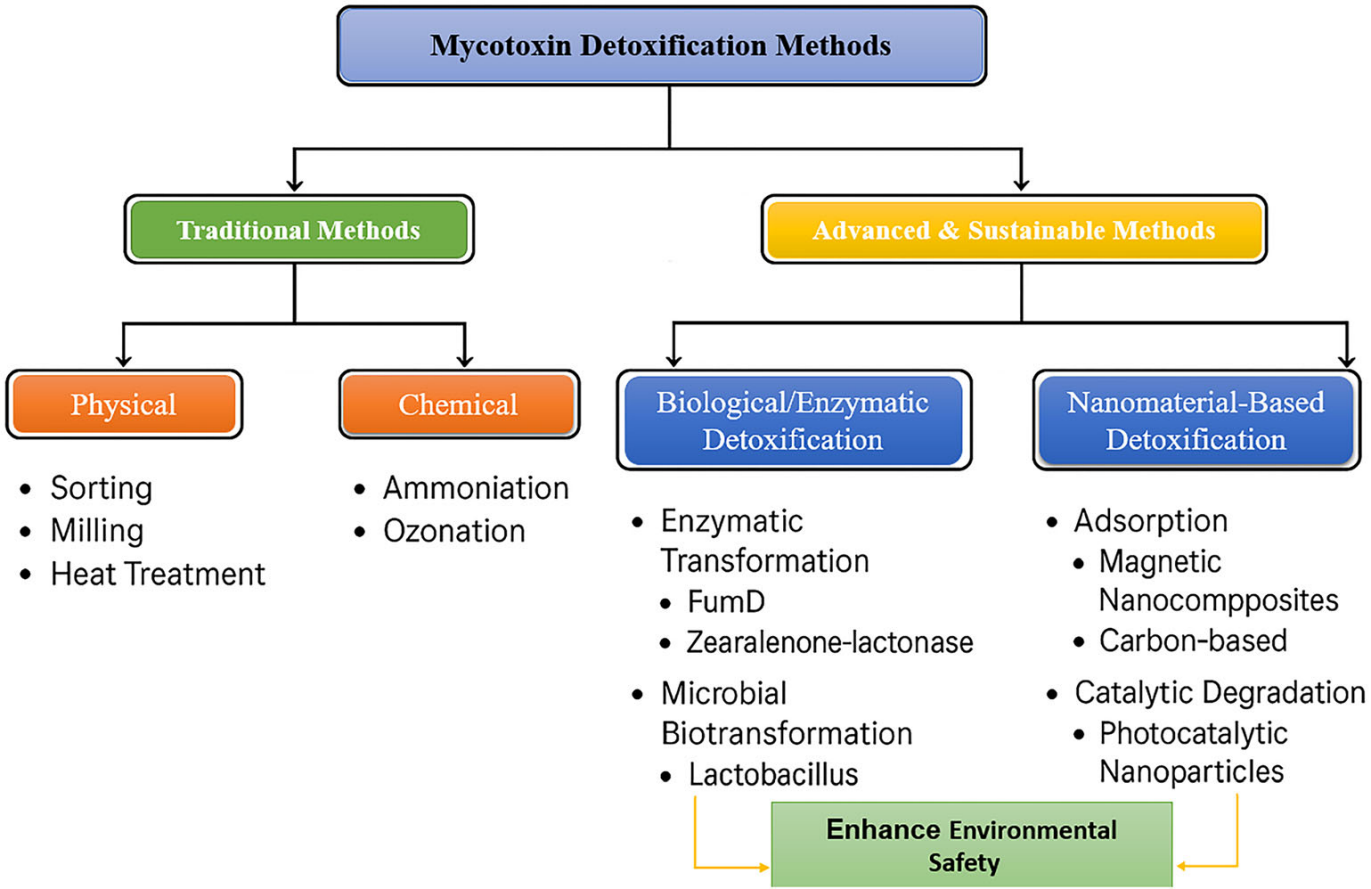
Schematic representation of mycotoxin detoxification strategies.

Traditional methods often prove insufficient since multiple mycotoxins exhibit resistance to heat, and chemical treatments can compromise the quality of the final product.^68^ These limitations have prompted a transition from conventional technologies to biologically and nanotechnology‐based methodologies, which provide more selective, efficient, and sustainable alternatives.^69^ Table 1 offers a comprehensive comparison of detoxification processes to elucidate the primary differences between these approaches.

**Table 1:** Comparative overview of traditional and advanced mycotoxin detoxification methods in cereal products

Method type	Method	Principle	Advantages	Disadvantages	Suitability	Ref.
Traditional methods	Physical (e.g. sorting, heat)	Removal or degradation of toxins through physical processes	Simple to implement; low cost for sorting; effective for reducing mycotoxin levels	Incomplete detoxification may negatively affect food quality and nutrients; many mycotoxins are heat‐stable	Smallholder farms, preprocessing stages	70, 71, 72
Traditional methods	Chemical (e.g. ammoniation)	Chemical degradation of toxin structure using reagents	High detoxification efficiency for specific mycotoxins (e.g. aflatoxins)	Potential for toxic residues; negative impact on food taste and nutrition; creates secondary pollutants	Industrial‐scale applications, animal feed	73, 74, 75
Advanced methods	Biological/enzymatic	Conversion of toxins to nontoxic or less toxic metabolites using microbes or enzymes	Highly specific; preserves food quality; environmentally friendly; reusable; capable of multi‐mycotoxin degradation	Slower process; challenges with scalability and cost; regulatory approval required for enzymes; may alter texture/flavor	Food and feed industries, research applications	13, 76, 77, 78
Advanced methods	Nanomaterial‐based	Adsorption or photocatalytic degradation of toxins using nanoparticles	High efficiency; large surface area; magnetic or photocatalytic properties enable easy removal; can be cost‐effective; multifunctional for detection and detoxification	Potential for nanomaterial residues in food; toxicity concerns of some nanoparticles; requires further research on safety	Postharvest processing, targeted detoxification	52, 53, 79

### Physical, chemical, and biological detoxification approaches

Physical detoxification techniques, including optical classification, milling, extrusion, heated steam, and cold plasma, decrease mycotoxin concentrations after harvest yet preserve food quality.^70^ Chemical detoxification using ammoniation, sodium‐containing reagents, ozone, or natural plant extracts can break down certain toxins but may affect food taste or safety.^73^, ^75^ Biological detoxification is regarded as the most promising method due to its high specificity, gentle processing conditions, and ecologically beneficial features.^80^ Enzymatic breakdown using enzymes that include fumonisin esterase (FumD), zearalenone‐lactonase (Zhd101p), and dye‐decolorizing peroxidase (BsDyP) accelerates the breakdown of many mycotoxins, while significantly reducing toxicity.^77^, ^81^

Microbial detoxification utilizing bacteria (such as *Lactobacillus*), yeasts, or fungi presents effective and environmentally friendly approaches for cereal‐based products.^13^, ^76^ The expression of recombinant enzymes and the process of enzyme immobilization significantly improve detoxification efficiency, allowing for potential direct applications in food and feed products.^78^

The future advancements emphasize the incorporation of advanced materials, very sensitive MS, AI, and portable biosensors for efficient, real‐time, multi‐target detection.^18^ Hybrid strategies that integrate nonthermal physical treatments with biological detoxification and nanomaterial adsorbents are gaining recognition as environmentally sustainable, effective, and multifunctional methods.^82^ Genetic modification and the development of crop resistance provide sustainable preventive measures against fungal contamination.^83^, ^84^ These integrated approaches have been developed to address the global demand for high‐throughput, accurate, and sustainable management of mycotoxins in cereal‐based foods.^85^ Table 2 presents an in‐depth analysis of the primary mycotoxin detoxification methods examined in this section, emphasizing their underlying principles, benefits, and drawbacks. Recent advances in analytical science have hastened the advancement of field‐deployable, high‐throughput, and industry‐friendly detection tools that were developed to further overcome the limits of traditional chromatographic platforms. The emphasis is on speed, portability, multiplex capability, and minimal preparation, which can lead to a wider range of usage across the cereal supply chain rather than being limited to understanding a particular matrix, point in the supply chain, or technique.^86^, ^87^

**Table 2:** Comparative overview of mycotoxin detection methods in cereal products

Technique/approach	Description	Advantages	Limitations	Ref.
Traditional chromatography	HPLC (UV, diode array detector (DAD), FL, MS), GC (electron capture detector (ECD), flame ionization detector (FID), MS), LC–MS/MS for multi‐mycotoxin analysis	High analytical accuracy and selectivity; well established for regulatory testing	Time‐consuming, expensive, requires skilled technicians, not suitable for large‐scale or on‐site uses	27, 28, 29, 30
Sample preparation (SPE, MycoSep®, QuEChERS)	SPE, immunoaffinity columns, QuEChERS with sorbents like C_18_, PSA, GCB	Enables trace‐level detection; simultaneous multi‐mycotoxin analysis; minimizes matrix effects	May require additional cleanup; complexity increases with the number of analytes	24, 31, 32
Immunometric assays	ELISA, TLC, FTIR spectroscopy, NMR spectroscopy	Rapid screening; simple operation	Expensive antibodies; lower accuracy than LC–MS/MS; limited for on‐site high‐throughput uses	25, 35, 36
Spectroscopic and imaging techniques	NIR, Vis–NIR, MIR, fluorescence, hyperspectral and multispectral imaging, SERS	Nondestructive; fast; minimal sample preparation; suitable for large batches; potential for online integration	High initial cost; requires chemometrics and AI for data analysis	22, 26, 46
Molecularly imprinted polymers and nanomaterials	MIPs, graphene oxide, gold nanowires, hybrid polymer–metal–organic nanomaterials	High selectivity and sensitivity; can mimic antibodies; signal amplification; multifunctional detection/detoxification	Requires complex fabrication; may be expensive for large‐scale use	51, 53
Advanced sample preparation	Magnetic SPE, UHPLC–MS/MS, QuEChERS for modified and emerging mycotoxins	Rapid, efficient, and compatible with multi‐mycotoxin detection	May require specialized nanoparticles or equipment	21, 23, 54
Physical detoxification	Optical sorting, milling, extrusion, superheated steam, cold plasma	Reduces mycotoxins postharvest; preserves food quality	Not always complete; may affect texture/flavor	70
Chemical detoxification	Ammoniation, sodium‐based reagents, ozone, and natural plant extracts	Effective for specific mycotoxins; can be applied postharvest	May alter palatability or nutrition; regulatory constraints	70, 73, 75
Biological detoxification	Enzymes (FumD, Zhd101p, BsDyP), microbes (*Lactobacillus*, yeasts, fungi), recombinant enzymes	High specificity; mild conditions; eco‐friendly; multi‐mycotoxin degradation	Scalability, cost, and regulatory approval; may alter texture/flavor	13, 76, 77, 78, 80
Emerging trends and future perspectives	AI, HRMS, portable biosensors, hybrid nanomaterials, nonthermal + biological treatments, crop resistance	Rapid, on‐site, high‐throughput detection; eco‐friendly; integrated detoxification; preventive strategies	High initial cost; implementation challenges in developing regions	18, 82, 83, 84

### Biological and enzymatic detoxification

Biological detoxification, which utilizes microorganisms and enzymes, is widely considered one of the most promising methods.^88^ This approach aims to transform mycotoxins into nontoxic or less toxic metabolites under mild conditions that do not affect the nutritional value of the food.^89^

#### Enzymatic transformation

Enzymes, such as FumD, may break down some mycotoxins quite well. For instance, Zhou *et al*.^90^ demonstrated that zearalenone‐lactonase displayed on nanocarriers degraded over 90% of ZEN within 2 h under mild conditions, offering a green and efficient enzymatic detoxification strategy. FumD can break down more than 80% of fumonisin B in maize in about an hour, making it safe to eat.^91^, ^92^ Zearalenone‐lactonase and other enzymes do the same thing by deactivating zearalenone, making it safe to eat.^90^

#### Microbial biotransformation

Several microorganisms, including bacteria and yeasts, may naturally detoxify mycotoxins.^93^ For example, various species of *Lactobacillus* and certain strains of *Aspergillus* are potential biological agents that could biotransform mycotoxins into less toxic compounds. This technique is becoming more popular as a safe, green alternative to chemical methods.^94^ Figure 2 displays a conceptual summary of the main detoxification methods, including physical, chemical, biological, and nanomaterials.

**Figure 2:**
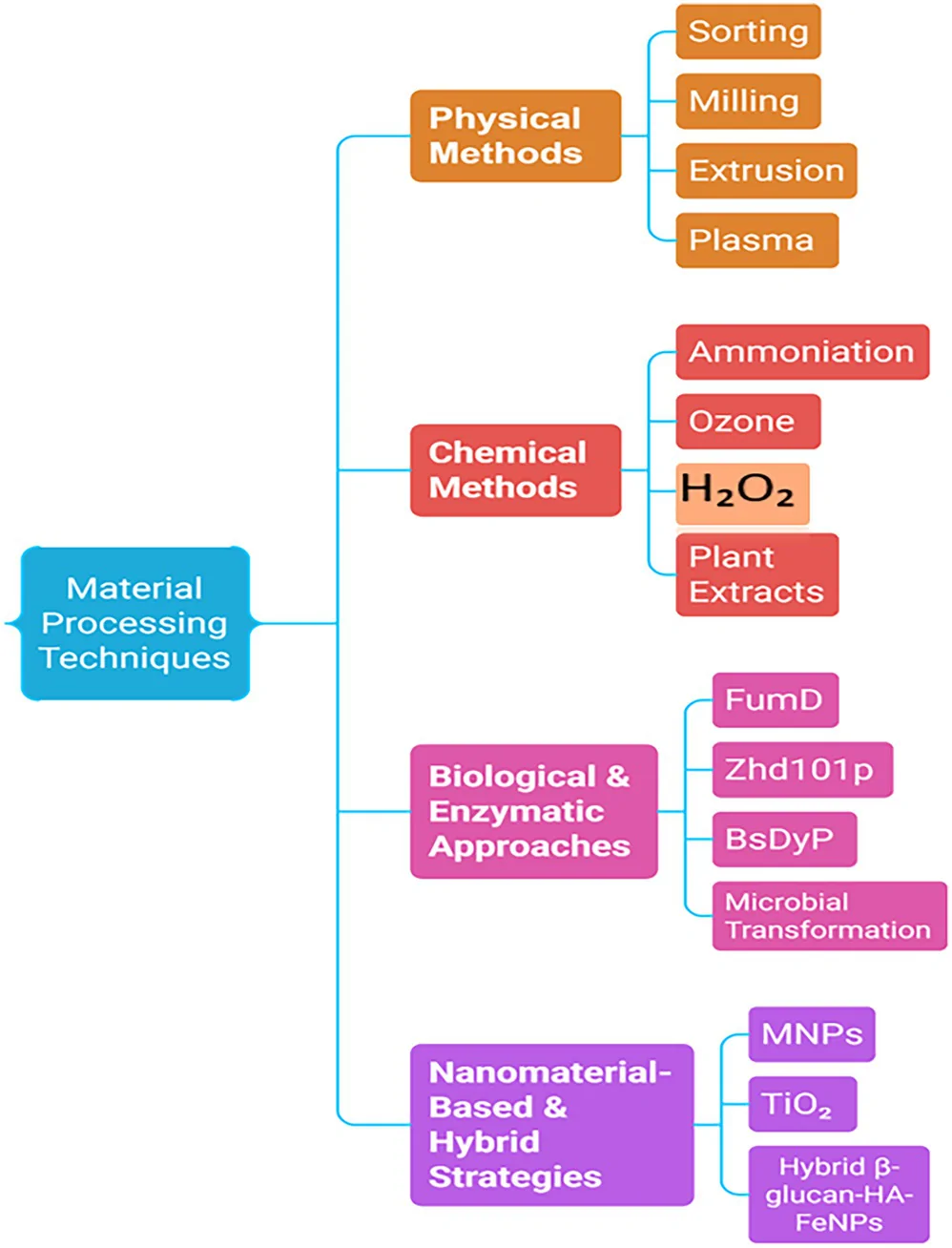
Overview of mycotoxin detoxification strategies.

### Nanomaterial‐based detoxification

Figure 3 shows that nanotechnology is a useful and strong technique for managing mycotoxins, mostly by adsorption and catalytic destruction. Nanotechnology is a potent and supplementary instrument to biological techniques, primarily via adsorption and catalytic degradation.^95^ Nanomaterials are highly effective at either catching or neutralizing mycotoxins because they have a lot of surface area and are very reactive.

**Figure 3:**
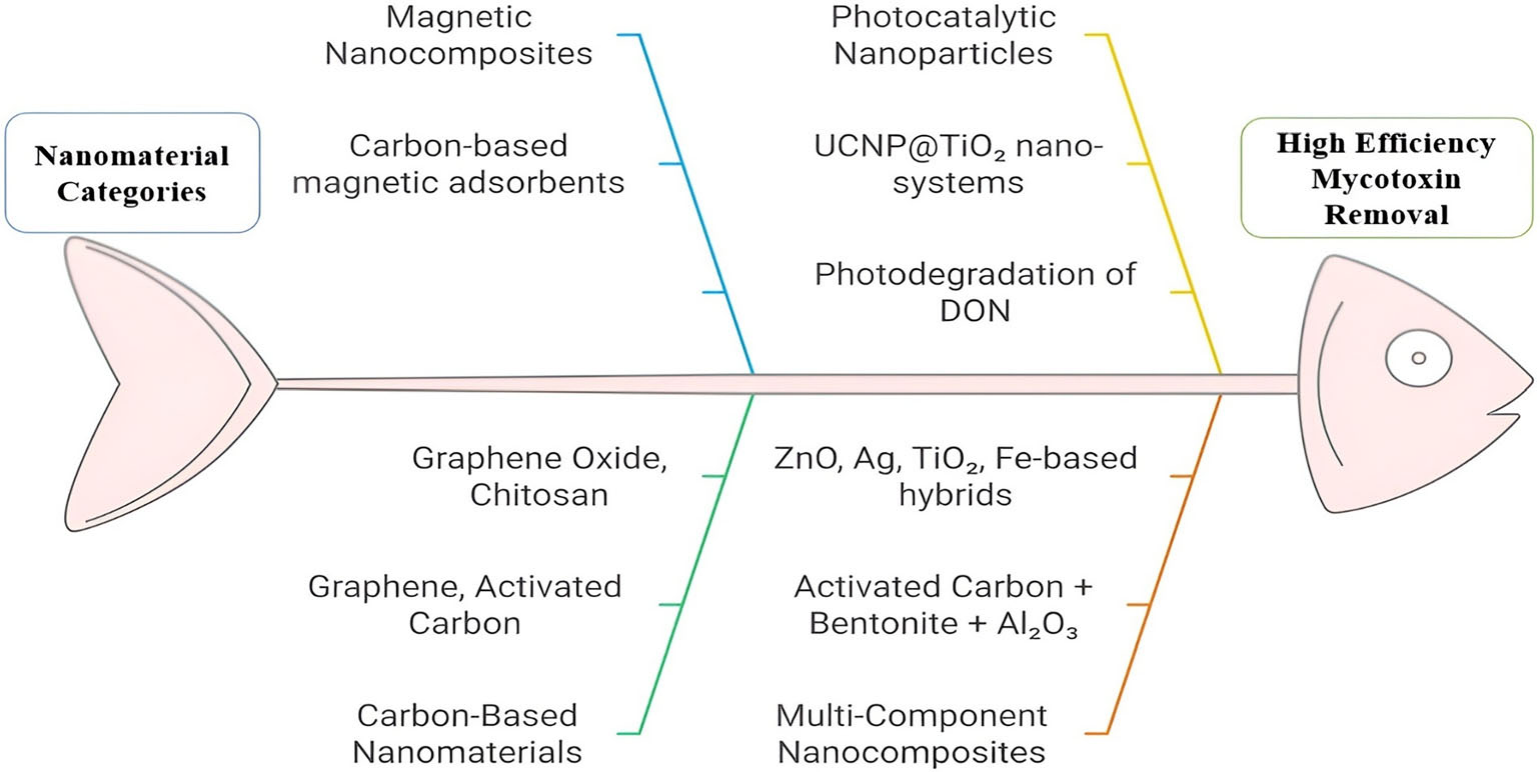
Categories of nanotechnology strategies for mycotoxin mitigation.

#### Nanocomposite adsorbents

Materials such as chitosan‐coated Fe₃O₄ nanoparticles and magnetic carbon nanocomposites exhibit high efficiency in mycotoxin adsorption.^96^ The advantages of their magnetic nature offer ease of removal from food matrices through the implementation of an external magnetic field, providing a simple method that involves less risk of recontamination.^13^

#### Photocatalytic degradation

This novel method involves the utilization of nanoparticles, including upconversion nanoparticle (UCNP)@TiO₂, for the degradation of mycotoxins under light irradiation.^97^ This method represents a ‘green’ and efficient method to fully degrade mycotoxins like DON, with the products being nontoxic.^98^, ^99^ Table 3 presents a comparative summary of the advantages, limitations, and applicability of both traditional and advanced mycotoxin decontamination strategies. Nevertheless, cereals are at risk of being recontaminated and of undergoing mycotoxin formation during the distribution and marketing processes unless proper storage and packaging solutions are implemented. Hence, ecological control and advanced packaging are considered as supporting pillars to detoxification‐based methods. Recent work by Adelusi *et al*.^100^ systematically optimized extrusion parameters (temperature, moisture content, screw speed, and residence time) and achieved over 80% reduction of multiple mycotoxins (aflatoxins, zearalenone, FUM, deoxynivalenol, and ochratoxins) in cereal‐based products while preserving nutritional quality.

**Table 3:** Comparative examples of advanced and traditional mycotoxin detection techniques

Method	Example LOD range	Approx. time/sample	Advantages	Limitations	Ref.
LC–MS/MS (UHPLC–MS/MS)	ng–μg per kg (depending on analyte/matrix)	Hours (including preparation)	High accuracy; multi‐mycotoxin; regulatory accepted	Time‐consuming; high cost	21, 30
Lateral flow immunoassay (LFIA)/rapid test strips	ng‐level (enhanced with nanoparticles)	Minutes	On‐site use; fast; user‐friendly	Semiquantitative; requires calibration	45
SERS (with QuEChERS pretreatment)	Reported down to sub‐μg kg^−1^	Minutes to 1 h	Very high sensitivity; minimal sample volume	Substrate standardization; variable reproducibility	22
QuEChERS + magnetic SPE	N/A (improves recovery, reduces matrix effects)	A few minutes (preparation)	Simultaneous multi‐analyte cleanup; cost‐effective	Requires matrix‐dependent optimization	23, 32

The table presents a summary of the fundamental concepts, analytical benefits, and practical limitations related to both traditional and advanced mycotoxin detection methods.

## ECOLOGICAL CONTROL AND STORAGE‐BASED PREVENTION

Mycotoxin management goes beyond detoxification; it also includes the storage and packaging of finished products to minimize the risk of recontamination and fungal growth.^101^ Programs that rely on traditional approaches, such as hermetic storage, provide conditions that are reasonably hostile to fungi, but the modern food safety landscape is pushing for a more proactive and intelligent solution. In response, active and intelligent packaging systems have emerged.^102^

### Primary prevention: ecological control in the field and storage

To effectively manage mycotoxins, one must begin with proactive primary prevention methods, well before even the processing stage. The significant ecological parameters associated with mycotoxins in cereals, preharvest and postharvest, are access to water (water activity, *a*_w_) and temperature.^103^ Spoilage fungi require specific combinations of these climatic factors for germination and toxin biosynthesis. Thus, approaches focused on controlling the storage environment are key.^104^ Low water activity and stable, low temperatures during storage are the best first defenses against mycotoxigenic fungi growth. Incorporating accurate information on these factors is a priority for the development of predictive risk assessment tools and management information specific to regions, especially as it relates to global climate change.^103^

### Active packaging systems

Active packaging extends beyond mere provision of a barrier by actively engaging with food products to enhance their shelf life and quality. Such systems are very effective in slowing down mold growth and, thus, mycotoxin production.^101^ The active packaging technologies actively engage with cereal products to deter the formation of microorganisms and slow down quality loss processes. They fundamentally employ two control methods of mycotoxins: moisture regulators that use moisture‐absorber agents and antimicrobial active packaging that uses carbon dioxide, ethanol, preservatives, inorganic nanoparticles, and plant extracts.^19^

Packaging films contain plant‐based essential oils like cinnamon and anise, as well as natural extracts and nanoparticles.^105^ The agents then gradually diffuse over a period of time to establish an antimicrobial environment that inhibits the growth of mycotoxigenic fungi like *Aspergillus* and *Penicillium*.^106^ Natural extracts, including bee bread and plant‐based essential oils such as cinnamon, anise, and orange, have been found to have antifungal activity when applied to packaging films.^107^, ^108^, ^109^ Films that included anise essential oil had significantly lower water vapor permeability and were found to prevent the growth of *Penicillium* sp. and *Rhizopus* sp. significantly better than control films without essential oils.^107^ Films with silver nanoparticles have been found to effectively limit fungal growth and aflatoxin contamination of grains.^13^ Kępka‐Borkowska *et al*.^109^ recently demonstrated that hermetic Purdue bags significantly reduced aflatoxin levels in stored maize by creating a CO₂‐rich environment that suppresses fungal growth, highlighting the potential of active packaging for postharvest mycotoxin prevention. *C*ontrol of moisture is necessary, as a higher relative humidity greatly aids fungus proliferation. The technologies of active packaging can employ moisture‐absorber chemicals that maintain a low‐humidity atmosphere and thus prevent mold and mycotoxin formation.^19^, ^101^

### Intelligent and smart packaging

Intelligent packaging systems improve safety through the provision of real‐time information on a product's condition.^110^ Such systems utilize internalized sensors to monitor key parameters and inform parties of potential danger.

#### Embedded sensors

Intelligent packaging includes sensors that measure characteristics like temperature and humidity, offering valuable real‐time information about the product inside. Other available systems include indicators (time–temperature, freshness, or gas indicators), sensors (chemical or biosensors, or edible sensors), and data carriers (barcode labels and RFID tags).^111^ Global positioning system (GPS) technology can also be integrated with smart packaging for traceability and real‐time location monitoring of cereal products during transit. The currently underdeveloped nanodiagnostic kits allow for use in the field to detect plant pathogens or disease mitigative measures and to utilize diagnostic tests for the detection of the most common mycotoxins at or below the EU maximum residue limits, allowing real‐time detection of mycotoxins on a single strip panel of cereals.^19^

#### Hermetic and smart packaging solutions

The best way to store cereal grains safely for an extended period is in hermetic packaging bags. In addition, packaging that has been designed to be antifungal using antifungal compounds, either enhanced or fortified with essential oils or organic acids, is generating scientific research related to pathogenic fungi.^101^ Purdue enhanced bags for crop storage are an instance of hermetic packaging. The literature indicates that this packaging significantly reduced aflatoxin levels in maize, likely by providing a hostile environment for fungi to colonize or develop toxins.^109^, ^112^, ^113^

#### Combining active and intelligent features

What these two ideas come together to form is smart packaging that not only protects a product but also indicates the safety of the product within.^114^ The use of these new technologies decreases fungal contamination, increases shelf life, and decreases waste, all while promoting sustainability.^115^ This method is aligned with the hazard analysis and critical control points (HACCP) system, as it provides continuous and simple technology to monitor feed and food safety.^19^ Figure 4 illustrates the critical control points for detection, monitoring, and intervention across the cereal supply chain, from preharvest to end use.

**Figure 4:**
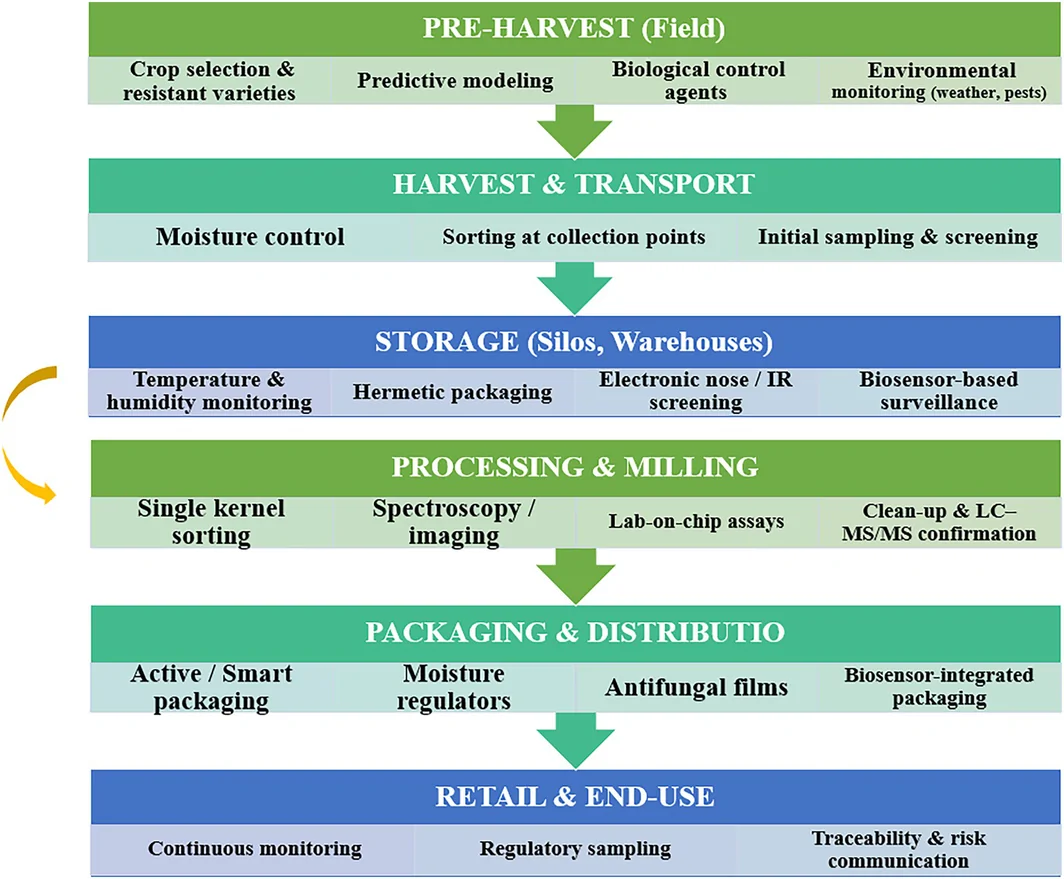
Supply chain control points for mycotoxin detection and intervention.

## FUTURE PERSPECTIVES

The issue of mycotoxin contamination in the cereal supply chain requires a comprehensive and multifaceted approach for its mitigation. As we have shown in this review, mycotoxin management will be aided by novel technologies that support and enhance the capabilities of conventional methods.^19^

### Technological convergence

#### AI and omics

HRMS with artificial intelligence will transform mycotoxin detection through enhanced sensitivity and accuracy, while metabolomics will allow for the detection of hundreds of compounds at once.^18^ AI can be put to use in numerous areas of mycotoxin management, including predictive modeling of mycotoxins and analytical detection and analysis.^86^

Several AI‐powered methods have been investigated within the last few years for the analysis and prediction of mycotoxins in cereals. Each technique possesses particular strengths and limitations. Table 4 presents the relevant details of various AI‐powered methods, including those based on deep learning (DL) approaches and hybrid methods as portable technologies, along with their target matrices and references.

**Table 4:** AI‐based approaches for mycotoxin detection and prediction in cereals

Detection/application	AI approach/model	Food matrix/target	Performance metrics	Ref.
Imaging‐based mycotoxin detection	CNN (GoogLeNet, SqueezeNet, AlexNet, ResNet50)	Various grains, nuts	High detection accuracy (>90%)	116, 117
Hybrid ML‐deep learning (CNN‐SVM, 3D‐CNN‐RF)	CNN + support vector machines (SVMs)/radio‐frequency (RF)	Cereal grains	Enhanced detection, multi‐feature integration	47, 118
Spectroscopy‐based ultra‐sensitive detection	1D and 2D CNN + SERS	Corn oil, zearalenone	Detection limit: 6.81 × 10^−4^ μg mL^−1^	119, 120, 121
HIS	HSI + SVM/RF/artificial neural networks (ANNs)	Cereals, nuts	Multi‐mycotoxin detection; classification accuracy up to 95.7%	47, 48
Field‐level wheat monitoring	YOLOv5 and DeepMAC + extreme gradient boosted trees (XGBoost)	Wheat ears (fusarium head blight)	Mean accuracy and F1 >89%	122
Pistachio contamination detection	ResNet + HIS	Pistachios	96.67% classification accuracy	49, 123
Portable real‐time detection	ISCA‐MobileNetV3 + artificial olfactory sensors	Maize	>98.5% accuracy; evaluation < 1 s	59
Portable electrochemical biosensors + IoT	ML algorithms + IoT	Corn, multiple mycotoxins	*In situ* multi‐mycotoxin quantification; GPS tracking	59, 124
Environmental monitoring	Arduino sensors + compact convolutional transformer	Wheat storage	83.33% accuracy for contamination prediction	59, 125
Cumulative learning for aflatoxin detection	CNN + transfer learning	Peanuts	Accuracy > 97%	126
Predictive mycotoxin management	Random forest, mechanistic models (AFLA‐PISTACHIO)	Corn, barley, pistachios	Up to 95.6% correct preharvest prediction	117, 123, 127
IoT‐enabled supply chain monitoring	AI + sensors + cloud computing	Grains, storage facilities	Continuous monitoring; early warning; optimal storage	47, 109

#### Bio–nano hybrid systems

Future studies should experiment with multienzyme cascades and cooperative degradation systems that not only take advantage of the specificity of the enzymes but also the advanced features of nanomaterials.^67^ This combination could provide a one‐pot, highly efficient method to simultaneously remove diverse mycotoxins.^128^

#### Integrated solutions

Ultimately, the goal is to establish seamless systems that significantly advance breeding for crop resilience, portable biosensors for rapid on‐site detection, and smart packaging that actively avoids contamination and provides real‐time quality data.^23^

### Addressing implementation challenges

Although progress is encouraging, there are still challenges to address. The cost associated with emerging technologies and their complexity limit their widespread adoption, especially in developing countries where mycotoxin contamination is a food safety issue.^13^, ^129^ In the future, agricultural and industrial stakeholders must implement affordable, scalable, and practical technologies.^130^ In addition, global collaboration will be critical for developing robust and standardized risk assessment tools and regionally relevant management guidance for this global food safety issue.

## CONCLUSIONS

Mycotoxins will remain a recurrent challenge for cereal‐based food systems, even as analytical and processing technologies continue to advance. The evidence reviewed in this paper shows that relying solely on traditional endpoint control measures is no longer sufficient. A supply chain‐oriented strategy that links three pillars, namely rapid and reliable detection, effective detoxification, and storage and packaging solutions to prevent recontamination, offers a more realistic and sustainable way forward for cereal grains. Across the studies assessed here, newer detection platforms based on advanced chromatography, spectroscopy, nanomaterials, and AI‐supported data analysis strengthen early warning and routine monitoring. At the same time, biological and enzymatic detoxification, supported by nano‐enabled adsorbents or photocatalysts, provides gentler and often more selective options than conventional physical and chemical treatments. Active and intelligent packaging systems add a final layer of protection by limiting fungal growth during storage and distribution and by supplying real‐time information on product status. However, the integration of such advancements into daily practice will depend on various factors beyond technology, including cost, regulatory approval, safety evaluation of new materials, and the enhancement of human resources in regions with the highest mycotoxin risk. Progress on these fronts, together with more harmonized risk assessment and international collaboration, will be essential if the full potential of these tools is to be realized and mycotoxin burdens in cereal supply chains are to be reduced durably.

## FUNDING INFORMATION

The authors would like to thank Deakin University for the funding support.

## CONFLICT OF INTEREST

The authors declare that they have no known competing financial interests or personal relationships that could have appeared to influence the work reported in this paper.

## AUTHOR CONTRIBUTIONS

Abolfazl Asqardokht‐Aliabadi: Conception, Method, First draft composition, and assessment; Editing. Dipendra Kumar Mahato: Writing, Reviewing, Editing, and Supervision. Jafar Mohammadzadeh Milani: Conception, First Draft Composition, Writing, Reviewing, Editing, and Supervision. The authors have read and agreed to the published version of the manuscript.

## Data Availability

The data that support the findings of this study are available from the corresponding author upon reasonable request.
